# Anomalous Affections of the Teeth

**Published:** 1859-04

**Authors:** J. Smith Dodge


### ARTICLE VI.

*Anomalous Affections of the Teeth.* By J. SMITH DODGE, Jr., M. D., D. D. S.

THE three following cases of a very uncommon affection, are the only ones of the kind I have seen or heard of. At the same time, although obtained from very different sources, they seem so perfectly to explain the progressive steps of the disease, that they naturally fall into close connection and definite order.

I. A left upper central incisor, of medium size, rather broad than thick, but apparently not of very strong texture, extracted from the mouth of a young man many years ago. Previous history unknown. The crown of the tooth is perfect, except that it has been filled in the centre of the labial surface; a pretty large filling for the position. The root is of about three-fourths its natural length, and excavated to a thin shell, so that the termination, irregularly oblique, is a sharp edge, except where it is thickened at one point by a small tubercle of cement. From this opening the cavity gradually narrows as it runs toward the crown, and at its extremity may be seen the inner surface of the filling mentioned above. The diameter of the excavation, at the end of the fang, is full two lines, and it cannot be much less where it is terminated by the filling. The irregularity of the edge, and the large size of the excavation, as well as the age of the patient and the wide exposure of the inner surface of the filling, preclude the idea of an arrest of development before the fang was fully formed.

II. Left upper central incisor, of much the same shape as the former, but of the strongest texture, and the crown entirely perfect except where it is worn away against the lower teeth. History entirely unknown. The root is of the full natural length and size, with the vascular opening at its extremity entirely normal. About two-thirds of the distance from the neck to the end of the fang, on the posterior aspect of the root, and to the right of its central line, is an opening about a line and a half long, and half the width; the length corresponding with the long diameter of the tooth. This is the orifice of a deep cavity, rather wider than the opening, and having at its bottom a slit by which it freely communicates through its whole length with the pulp-canal. The root is a little enlarged by cement about the orifice described. The pulp-canal does not seem enlarged at this point.

III. Left upper central incisor; crown longer than in the others, and of rather an opaque color, but perfectly sound, and well worn against the lower teeth. Extracted recently from the mouth of a lady some forty years of age, far advanced in phthisis. The tooth had never been very troublesome, but was extracted to prepare the mouth for a suction plate. The root is irregular, being somewhat thickened by cement, roughened by absorption, and darkened by a thin layer of rough tartar at some points. Length normal. Vascular canal at apex, natural. About half way from neck to apex, partly on the right and partly on the anterior surface of the root, and lying a little obliquely to the axis of the tooth, is an opening similar in shape, but not quite equal in size, to the one in the preceding case. The cavity to which this opening leads is neither so deep nor so wide as in the former tooth, nor does it open into the pulp-canal. But at the two ends of the bottom of this cavity, are two small openings, into which a fine bristle may be passed in the direction of the pulp-canal, but it is suddenly arrested at about half way.

When I first saw the first of the specimens described above, I was convinced that the tooth must have been originally completely formed, and that the excavation could only have resulted from an immense hypertrophy of the pulp, which had caused here, as aneurismal tumors often do, the absorption of the bone which impeded its expansion. I then supposed this hypertrophy to have affected the normal vessels of the pulp; and this may still have been the case, but the two other examples, and the obliquity of the large orifice, make me suspect that in this instance, as in the others, the diseased action may have begun on the side of the root, and extended toward the apex until it implicated the normal orifice and the vessels which naturally occupy it. In the second and third cases, I conceive the morbid action to have begun in one of those abnormal vessels which are known sometimes to penetrate the fangs of teeth, connecting the pulp with the periosteum of the root. In the third case there seems to have been two of these, or one which branched. This action seems, too, to have begun on the surface of the root, as well as in the first case—for the orifice is larger than the internal diameter—as in the last two; and the evidence which the surface of the root bears, in Case No. 3, of general irritation of the covering membrane, would seem to indicate that the diseased action in question was only a local increase of the malady which affected the whole periosteum. That such a local action once inaugurated should continue indefinitely, can be no matter of wonder, for these slow and protracted local affections try the utmost skill of the surgeon daily. But it certainly is rare to find in these organs, placed on the very outposts of the system, so slow in their vital changes, and so prone to withdraw their more delicate tissues behind a rampart of new formed bone, manifestations of such active vitality.

If my conjectures are well founded, each of these cases has begun about an abnormal vessel; and it may be that the affection cannot occur about the normal vessels entering at the apex, because in that case the disease of the periosteum, (beginning at the neck, from inflammation of the gums, or tartar,) would necessarily be so great as to cause the speedy expulsion of the tooth; while a much less degree of general irritation might implicate a vessel emerging half way down towards the neck.

That all my cases should be in the same tooth, is, I suppose, only a coincidence.

